# Relationship of the type of breastfeeding in the sexual function of women*

**DOI:** 10.1590/1518.8345.3160.3438

**Published:** 2021-07-19

**Authors:** Juliana Bento de Lima Holanda, Solina Richter, Regiane Bezerra Campos, Ruth França Cizino da Trindade, Juliana Cristina dos Santos Monteiro, Flávia Azevedo Gomes-Sponholz

**Affiliations:** 1 Universidade de São Paulo, Escola de Enfermagem de Ribeirão Preto, PAHO/WHO Collaborating Centre for Nursing Research Development, Ribeirão Preto, SP, Brazil.; 2 Faculty of Nursing, Univerasity of Alberta, Edmonton, Alberta, Canada.; 3 Professor and Academic Director, Global Nursing Office.; 4 Universidade do Oeste do Paraná, Centro de Educação Letras e Saúde, Foz do Iguaçu, PR, Brazil.; 5 Scholarship holder at the Fundação Araucária, Brazil.; 6 Universidade Federal de Alagoas, Escola de Enfermagem e Farmácia, Maceió, AL, Brazil.

**Keywords:** Breast Feeding, Sexuality, Postpartum Period, Public Health, Cross-Sectional Studies, Women’s Health, Aleitamento Materno, Sexualidade, Período Pós-Parto, Saúde Pública, Estudos Transversais, Saúde da Mulher, Lactancia Materna, Sexualidad, Período Posparto, Salud Pública, Estudios Transversales, Salud de la Mujer

## Abstract

**Objective:**

to relate the type of breastfeeding in the women’s sexual function.

**Method:**

a cross-sectional study conducted with 150 women in the postpartum period registered in the Family Health Strategy of a large Brazilian municipality. Two instruments were used: one for characterizing sociodemographic, obstetric and breastfeeding variables, and the Female Sexual Function Index for the sexual function. Descriptive data analysis was performed, comparing the variables of interest using the Analysis of Variance, Brown-Forsythe and Tukey tests.

**Results:**

there was statistical significance between the groups that practiced different types of breastfeeding in the vaginal lubrication domain (p = 0.015), with the mothers in mixed or partial breastfeeding presenting a higher score for this domain (3.8).

**Conclusion:**

there is a difference in the female sexual function between different types of breastfeeding. Women who presented better vaginal lubrication belonged to the mixed breastfeeding group.

## Introduction

As a social and multidimensional practice, breastfeeding involves several aspects of the woman’s life, the mother-child binomial, the family and the couple, including sexual behavior^1^. Like breastfeeding, sexual response results from complex interactions between biological and psychosocial factors, which vary among cultures, individuals, depending on time, environment and circumstances^2^.

Some couples can experience sexuality concomitantly with breastfeeding in a positive or negative way, according to the sexual response and possible interaction between the couple. Sexual dysfunctions are frequent during breastfeeding and are defined as disorders related to obtaining sexual desire and satisfaction^3^.

There is limited information on the incidence and prevalence of female sexual dysfunction^4^. The available data differ due to variations in the definitions of sexual dysfunction, composition of the samples in the research studies, methods of data collection, and the way the instruments were validated^4^. In this sense, the studies show a wide prevalence of female sexual dysfunction, which has been obtained in different ways. Considering the variety of existing assessment methods, there is international consensus that the prevalence of female dysfunction, regardless of age, is 40% to 50%^2^^,^ 5. When considering the pregnant puerperal cycle, recent studies show a high prevalence of sexual dysfunction ranging from 41% to 83% in the first three months^6^ and around 60% in the first postpartum year^3^^,^ 7.

Thus, the perinatal period is characterized by a decline in sexual activity^3^, in which there are reports of higher levels of sexual dysfunction and reduced sexual desire. Breastfeeding women report sexual inactivity or dysfunctional problems more often^8^. During breastfeeding, hormonal changes occur that involve the secretion of prolactin and androgen receptors, suppressing the libido and interfering with the sexual response phases^9^. Other aspects of motherhood, such as night sleep deprivation and baby care, may come to interfere with the woman’s sexual response cycle. These physical, hormonal, and social changes involved in breastfeeding are well described in the literature. However, it was sought to know if the time and type of breastfeeding influence the woman’s sexual function. Despite the significant impact on life, the sexual function of women after childbirth is often neglected by the health professionals^4^. Perceiving that the female sexual response suffers hormonal and emotional interference in the postpartum period, which can manifest itself in sexual dysfunctions, and recognizing that sexual dysfunction can influence early weaning, is of paramount importance for the nurses who assist nursing mothers.

This study aims to relate the type of breastfeeding to women’s sexual function.

## Method

This is an observational study with a cross-sectional design, conducted with 150 nursing mothers recruited from Family Health Strategy units in the city of Maceió, AL, Brazil, in 2017.

The reference population consisted of all adolescent and adult women who were breastfeeding their children, regardless of the type or duration of breastfeeding, as long as they were established in the breastfeeding categories of the World Health Organization and of the Ministry of Health.

The participants were selected according to the following inclusion criteria: women between three and six months postpartum, who were breastfeeding, who had a sexual partner, and who had resumed sexual activity after delivery. The exclusion criteria were as follows: women with any health condition that contraindicated sexual activity, women from seven months postpartum, and women who use drugs or other psychoactive substances, with a history of psychiatric illness or chronic diseases, such as cancer and neurological diseases, as these factors negatively interfere in one or more phases of sexual response^10^.

The calculation of a simple random sample was performed based on the prevalence of exclusive breastfeeding in children under six months of age in the municipality, obtained from the II National Survey on the Prevalence of Breastfeeding in the Brazilian Capitals and the Federal District, of 34%. A tolerable sampling error of 5% was considered, as well as a 95% confidence level, and an expected loss of 10%. The calculated sample was 150 participants.

Two instruments were used for data collection. The first instrument was developed based on the professional experiences of the authors and after reading national and international scientific publications on the theme. With 14 structured questions, it contained sociodemographic variables of the nursing mothers (age, religion, schooling, work, profession, and family income), obstetric (postpartum time, number of parturition, return to menstruation and cesarean section as a way to resolve the last pregnancy) and type of breastfeeding practiced.

The information on the type of breastfeeding practiced followed the definitions of the World Health Organization and of the Ministry of Health and included exclusive, predominant, complemented and mixed breastfeeding^11^. The criteria for characterizing the types of breastfeeding in this research followed the definitions of BF adopted by the World Health Organization and recognized worldwide^11^.

Exclusive breastfeeding: when the child receives only breast milk or human milk from another source, without other liquids or solids.

Predominant breastfeeding: when the child receives, in addition to breast milk, water or water-based drinks, fruit juices and ritual fluids.

Mixed breastfeeding: when the child receives breast milk and other types of milk.

Complemented breastfeeding: when the child receives breast milk and any solid or semi-solid food for the purpose of supplementing it, not replacing it.

The second data collection instrument was the Female Sexual Function Index (FSFI), a questionnaire that assesses women’s sexual health. This instrument was developed in the United States, validated and adapted for Brazil^12^. The questionnaire contains 19 questions that assess sexual activity in the last four weeks, divided into six domains: desire, excitement, vaginal lubrication, orgasm, satisfaction and pain, where each has a score and the total score refers to the sum of the scores multiplied by their respective factor. If the total value is less than or equal to 26.55, it indicates that the participant has some type of sexual dysfunction^12^.

The recruitment of the participants took place from routine home visits made by Community Health Agents (CHAs) of the FHS, in which the researcher accompanied the CHAs, in order to, at the end of the visit, present the research and make the invitation to the women. The possible participants, identified by the established criteria, were invited to participate in the research, and informed that they could choose another day for the interview or that it could be done immediately. After being aware of the research and of the ethical aspects, the women who agreed to participate signed the Free and Informed Consent Form (FICF). When the possible participant was identified as under 18, the presence of the legal guardian for the adolescent was requested, and the study was presented to this person. After the agreement and expression of the adolescent’s desire to participate, the Free and Informed Assent Form was signed by them, as well as the FICF by the legal guardian. Data collection took place from May to November 2017, at the participants’ homes.

A group of four nurses was created and trained by the main researcher to conduct the interviews and use the data collection instruments, thus ensuring that all data were collected in the same way, following the same understanding of what was being surveyed. The four interviewers accompanied the Community Health Agents during the routine home visits in order to conduct the interviews, so as to ensure that there was no doubt by the participants about what was being asked.

A database was built with the aid of the Excel software, in which the sociodemographic variables, and those related to breastfeeding and to sexual function were inserted, according to the answers to the FSFI questionnaire.

For comparing categories of interest, the test used was the Analysis of Variance (ANOVA). For using this test, we verified if, for each variable (scales and dimensions), the variances were homogeneous between the groups (assumption for use). When the homogeneity of the variances was not verified, we adjusted them using the Brown-Forsythe (BF) test. In situations where there was a significant difference between the groups, in order to identify which categories differed, multiple comparisons were made (comparisons between two to two categories), using the Tukey test, or the Dunnett test, the latter when Brown-Forsythe (BF) adjustment was required. For all the comparisons, we considered a 5% significance level. Thus, we verified a difference between the groups when p-value<0.05.

In relation to the ethical aspects, the content of the Free and Informed Consent Form was read together with each participant, as well as the study objectives. The confidentiality of the interviews was guaranteed and the freedom to participate or not was granted, as well as to withdraw at any time, without any prejudice to their assistance in the referred Family Health Units. The study was approved by the Research Ethics Committee linked to the National Research Ethics Committee of the National Health Council with CAAE protocol No. 62265816.2.0000.5013.

## Results

Of the 150 women investigated, age ranged from 14 to 43 years, mean of 24.8 ± 6.4 years old and a median of 24.0. Among the other sociodemographic characteristics, 60 (40%) were evangelical, 68 (45.3%) had incomplete primary education, and 85 (56.7%) worked as housewives. The monthly family income varied from one to two minimum wages for 81 (54.0%) of the families.

Women were, on average, 4.3 months postpartum ± 1.2 months; 91 (60.7%) had given birth more than once, 83 (55.3%) had already returned to menstruating, and 72 (48.0%) underwent cesarean section as a way to resolve the last pregnancy.

At the time of data collection, the breastfeeding groups found were exclusive, predominant, complemented and mixed breastfeeding. Regarding the duration of breastfeeding, 48 (32%) women breastfed for five months or more and 60 (40%) breastfed their children for less than four months, regardless of the type of the breastfeeding practiced (Table 1).

**Table 1:** Distribution of the participants according duration and type of breastfeeding practiced (n=150). Maceió, AL, Brazil, 2017

Variable		n	%
Duration of breastfeeding in months	< 4*	60	40.0
	4 - 5	42	28.0
	≥ 5	48	32.0
Type of breastfeeding	Exclusive	42	28.0
	Predominant	30	20.0
	Complemented	18	12.0
	Mixed	60	40.0

As for the breastfeeding time, no association was found between the occurrence of female sexual dysfunction and the duration of breastfeeding, even when each domain was analyzed separately (p>0.05).

Table 2 shows the type of breastfeeding and its influence on the female sexual function.

**Table 2:** Comparison of the mean of the sexual function scores and the domains according to the Female Sexual Function Index scale between the types of breastfeeding practiced. Maceió, AL, Brazil, 2017

Type of breastfeeding					
Sexual function	Exclusive Mean (SD*)	Predominant Mean (SD)	Complemented Mean (SD)*	Mixed Mean (SD)	p-value
Desire	2.9 (1.5)	3.2 (1.4)	2.9 (1.2)	3.4 (1.3)	0.274^†^
Excitement	3.8 (1.5)	3.7 (1.4)	3.8 (1.4)	4.2 (1.5)	0.335^†^
Lubrication	3.7 (0.5)	3.6 (0.4)	3.5 (0.4)	3.8 (0.6)	0.015^‡^
Orgasm	3.9 (0.9)	3.7 (0.9)	3.8 (0.8)	4.0 (0.9)	0.578^†^
Satisfaction	4.9 (1.4)	5.0 (1.3)	5.1 (0.5)	4.8 (1.4)	0.672^†^
Pain	2.8 (1.6)	3.0 (1.5)	2.6 (0.9)	2.5 (1.5)	0.330^†^
Total	22.0 (3.8)	22.3 (3.3)	21.6 (3.5)	22.7 (4.0)	0.723^†^

* SD = Standard Deviation;

† p -value obtained by means of the Analysis of Variance test;

‡ p-value obtained by the Brown-Forsythe test

There was no significant difference between the four types of breastfeeding practiced for the mean total score of sexual function between three and six months postpartum (p>0.05). The highest mean score of sexual function was 22.7 ± 4.0 and belonged to the group of women who practiced mixed breastfeeding.

**Figure 1:**
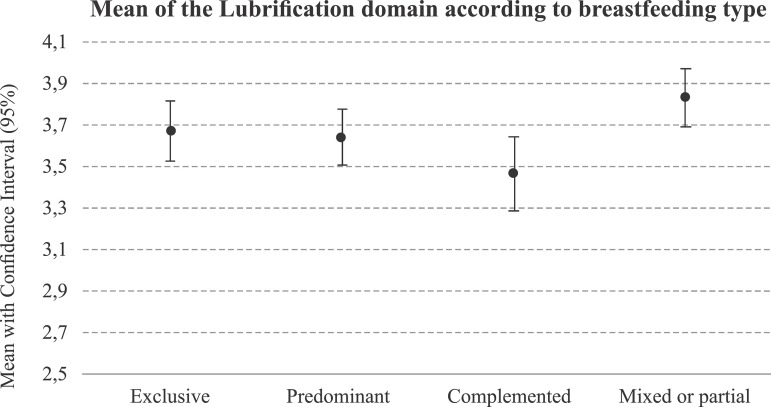
Analysis of the mean of the vaginal lubrication domain according to the type of breastfeeding practiced. Maceió, AL, Brazil, 2017

Regarding the analysis of the domains that make up the FSFI and the four breastfeeding groups investigated, there was a significant difference between vaginal lubrication (p = 0.015) and, among these women, those who practiced mixed breastfeeding (3.8 ± 0.6) presented the highest scores. When comparing the means of the vaginal lubrication domain, the women who practiced complementary breastfeeding presented a lower mean in this domain, as shown in Figure 1.

Multiple comparisons were also made between the breastfeeding groups and the vaginal lubrication domain, as shown in Table 3.

**Table 3:** Analysis of the multiple comparisons between the four types of breastfeeding and the vaginal lubrication domain. Maceió, AL, Brazil, 2017

Reference group	Comparison group		p-value*
Exclusive	x	Predominant	1.000
Exclusive	x	Complemented	0.423
Exclusive	x	Mixed or partial	0.529
Predominant	x	Complemented	0.582
Predominant	x	Mixed or partial	0.290
Complemented	x	Mixed or partial	0.018

* p-value obtained by means of the Tukey test

The results of the multiple comparisons above attest to the difference between the groups with supplemented breastfeeding and mixed breastfeeding (p<0.05), which demonstrates that there was statistical significance (p=0.018) in both groups.

## Discussion

This study was conducted to assess postpartum sexual function among women who practiced different types of breastfeeding. In the sample studied, the tendency to compromise sexual function was observed, with identification of low mean FSFI scores (<26.55), regardless of the type of breastfeeding, similarly to other studies^13^^-^ 14.

The highest mean score of sexual function (22.7 ± 4.0) was detected in the group of women who practiced mixed breastfeeding. These findings differ from an Iranian study, which detected a higher mean score of sexual function (23.6 ± 5.3) in women who practiced exclusive breastfeeding, in the period of four months after delivery^13^.

Breastfeeding causes different sensations and feelings in women in relation to their sexuality^15^. The positive effects of breastfeeding on the maternal sexual function are reported^13^^,^ 16, possibly due to increased breast sensitivity and increased oxytocin levels^14^. However, there are reports of negative effects of breastfeeding on the female sexual function, including fewer sexual relationships, low sexual desire, and low sexual satisfaction of women and their partners^15^^,^ 17 ^-^ 18.

It was identified that the women on mixed breastfeeding had more vaginal lubrication (3.8 ± 0.6) than those who practiced other types of breastfeeding; while another study detected greater lubrication in women on complementary breastfeeding (3.5 ± 1.0)^13^. Contrary to the findings of this study, a recent study on breastfeeding and sexual function, in primiparous women, found a significant relationship between low vaginal lubrication, dyspareunia and low desire in sexual activity in the presence of breastfeeding, in relation to the women who did not breastfeed, in the six-month period after delivery^19^. Considering the FSFI domains, it is noted that breastfeeding affects the desire, excitement, lubrication and pain domains^14^. In Italy, a study conducted with 269 women also measured sexual function through the application of the FSFI questionnaire and identified that those who were breastfeeding presented lower vaginal lubrication^8^. However, these studies do not specify the type of breastfeeding practiced.

When data between breastfeeding and non-breastfeeding women are compared, the results show that those who are breastfeeding are more likely to experience pain during intercourse and poor vaginal lubrication^6^^,^ 15. A possible justification for the low vaginal lubrication in breastfeeding women can be the physiological absence of the estrogen hormone during breastfeeding^6^.

In the case of women who are on mixed breastfeeding, other foods are offered for the baby, which increases the interval between feedings, induces ovarian cycles and the resumption of a woman’s hormonal physiology outside the pregnant-puerperal cycle, increasing the libido and vaginal lubrication. In fact, a higher sexual function score is observed in breastfeeding women who have resumed ovarian cycles, compared to women in lactational amenorrhea^16^. In addition to breastfeeding, among other factors associated with the lack of vaginal lubrication, dissatisfaction with body image stands out, especially in relation to overweight and obesity, reducing sexual interest^19^.

The influence of breastfeeding on the female sexual function must be considered, with the possibility of decreased vaginal lubrication, in addition to other changes, which can cause discomfort for the woman and consequently favor the reduction in the rate of exclusive breastfeeding. It was observed that exclusive breastfeeding up to six months, recommended by the World Health Organization^20^, was not practiced by part of the study participants, with interruption of breastfeeding at less than four months in 60 (40%), as well as early initiation of mixed or partial breastfeeding in 60 (40%), which reinforces the innumerable challenges for nurses and other health professionals, in order to implement public breastfeeding policies in the national territory and pro-breastfeeding practices^21^.

The importance of Nursing interventions is highlighted, with care centered on women^19^ and with the development of health education strategies in prenatal care, in order to discuss and contextualize the exercise of sexuality in the pregnancy-puerperal cycle, and its relationship with breastfeeding^14^. In addition, the participation of the partners in the puerperal consultations must be stimulated, in order to guide them in this regard, and the participation of the partner in prenatal care is also very important^15^^,^ 22.

Positive effects on the marital relationship were found in women who breastfed, for up to four months or for a period equal to or greater than five months, in relation to those who never breastfed, indicating that breastfeeding increases the levels of quality in the marital relationship throughout time, from the woman’s perspective. However, the duration of breastfeeding did not have the same influence for the partners. The results suggest that the improvement in the quality of the intimate relationship can be another psychosocial benefit experienced by the nursing mothers^23^.

Lack of information leads women to feel guilty and responsible for the loss of sexual interest^19^. The lack of sexual interest in the puerperal period, especially in the child’s first year of life, is a common event and occurs due to the transition from parenting and the definition of new roles, with a significant impact on family dynamics^19^. During the breastfeeding phase, the woman dedicates more time to the child and the perception of her body is directed as a source of nutrition, to the detriment of pleasure^15^.

From the guidelines and care centered on the women, they can get around the difficulties well and even strengthen their marital relationships, increase their self-esteem, sexual satisfaction and feel safe and empowered about breastfeeding and exercising their sexuality in this stage of the life cycle.

The results of this study contribute to the clinical practice, involving direct care for women and their families, groups and communities, by supporting reflections on the professionals responsible for the care of nursing mothers, for the development of advanced Nursing practices^24^, for promoting, protecting and supporting breastfeeding and the sexual and reproductive health of breastfeeding women. In this context, the Nursing workforce must be valued, as it has the capacity to fill gaps and unmet needs for care, especially in the context of primary health care^25^, with an impact on improving the health indicators of the population.

As this is an intimate forum issue, it is possible that some women did not fully report the truth about their sexuality, which is a limitation of the study. To reduce the bias, the interviews were conducted at the participants’ homes, a place that the women know and where they feel more comfortable, in a private and timely manner, according to her preferences and availability, and by prior appointment. The subjectivity of the term “vaginal lubrication” also stands out, which, during the application of the questionnaire, had a greater chance of personal interpretations, as to its meaning; to facilitate the approach to this question, popular terms were used for a better understanding of the aspect to be evaluated.

The evaluation of the woman during the gestational period, as well as at different points of observation in the puerperium, could provide a more detailed view regarding the study question. To advance this knowledge, it is recommended to carry out other studies, with a longitudinal approach, as well as the evaluation of women who do not breastfeed.

## Conclusion

There was a difference in the female sexual function between different types of breastfeeding. The highest sexual function score was found in women on mixed breastfeeding, as well as better vaginal lubrication. Exclusive breastfeeding was observed up to the period of six months of postnatal life, it was not being practiced by a portion of the women evaluated. The development of strategies for promoting, protecting and supporting breastfeeding, as well as the promotion of the sexual and reproductive health of the breastfeeding woman is recommended in order to clarify possible doubts, include the partner, empower women, and prevent the occurrence of undesirable events, considering the countless benefits of breastfeeding and sexuality on women’s health and well-being.
